# Supplementary material for: Efficacy of Low-Dose Prophylactic Quetiapine on Delirium Prevention in Critically Ill Patients: A Prospective, Randomized, Double-Blind, Placebo-Controlled Study
Source: J Clin Med. 2019 Dec 27;9(1):69. doi: 10.3390/jcm9010069 (PMC7019813; doi:10.3390/jcm9010069)
Supplement: Supplementary file 1 [file jcm-09-00069-s001.pdf]

Table S1. Rescue medication

Variables	Placebo group (N=20)	Study group (N=15)	p value
Total dose			
Remifentanil (mg)	16.54±16.92	9.81±17.66	0.261
Dexmedetomidine (mcg)	1784.03±2354.07	1482.83±3325.28	0.755
Midazolam (mg)	168.93±428.59	74.87±191.58	0.435
Average dose			
Remifentanil (mg/day)	2.75±2.40	2.30±3.40	0.652
Dexmedetomidine (mcg/day)	304.57±358.54	367.22±646.43	0.717
Midazolam (mg/day)	25.21±61.34	11.89±27.73	0.440
Other sedative drugs			
Haloperidol	3 (15.0)	0 (0.0)	0.117
Other drugs for delirium[a]	6 (30.0)	3 (20.0)	0.503

Note: Data are expressed as number (percentage) or mean ± standard deviation

[a]Other drugs for delirium included lorazepam or oral anti-psychotics

Table S2. Previous randomized controlled trials of pharmacologic delirium prophylaxis in ICU

Drug	Country	Study design	Setting	Cases	Primary outcome	Main results
Risperidone [19] 2007	Thailand	Prospective, double-blind, RCT	Cardiac surgery patient	Study: 63<br>Placebo: 63	Incidence of postoperative delirium	Reduced<br>(11.1% vs. 31.7%, p=0.009, RR=0.35)
Rivastigmine [18] 2009	Swiss	Prospective, Double-blind, RCT	Cardiac surgery patient	Study: 59<br>Placebo: 61	Postoperative delirium within 6 days	Failed<br>(30% vs. 32%, p=0.8)
Haloperidol [15] 2005	Netherland	Prospective, Double-blind, RCT	Hip surgery Patients	Study: 212<br>Placebo: 218	Incidence of postoperative delirium	No efficacy<br>(15.1% vs. 16.5%, RR=0.91)
Haloperidol [14] 2012	China	Prospective, Double-blind, RCT	Non-cardiac Surgery patients	Study: 229<br>Placebo: 228	Incidence of delirium within 7 days after surgery	Decreased<br>(15.3% vs. 23.2%, p=0.031)
Haloperidol<br>Ziprasidone [16] 2010	USA	Prospective, Double-blind, RCT	Medical ICU<br>Surgical ICU<br>Trauma ICU	Haloperidol: 35<br>Ziprasidone: 30<br>Placebo: 36	Number of days without delirium or coma	Feasible<br>(14.0 vs. 15.0 vs. 12.5 days, p=0.66)
Haloperidol [39] 2017	Japan	Prospective, open-label randomized trial	Postoperative elderly patients (>75yrs)	Study: 101<br>Placebo: 100	Incidence of severe postoperative delirium	Reduced<br>(18.2% vs. 32.0%, p=0.002)
Haloperidol	Netherlands			1 mg haloperidol: 350	28 days survival	No difference

[17]2018		Prospective, Double-blind, RCT	Medical ICU Surgical ICU Trauma ICU	2 mg haloperidol: 732 Placebo: 707		
Dexmedeto-midine [20] 2016	China	Prospective, Double-blind, RCT	Non-cardiac Surgery patients	Study: 350 Placebo: 350	Incidence of postoperative delirium	Decreased (9% vs. 23%, p<0.001, RR=0.35)
Dexmedeto-midine [21] 2018	Canada, US	Prospective, double-blind, RCT	medical-surgical ICU	Study:50 Placebo: 50	Proportion of patients who remained free of delirium	Reduced delirium Greater proportion of patients who remained delirium-free during the ICU stay (80% vs 54%, p=0.006 RR, 0.44)

Abbreviations: ICU, intensive care unit; RCT, randomized controlled trial; RR, relative risk
